# Inertial microfluidic mixer for biological CubeSat missions

**DOI:** 10.1007/s00604-024-06726-1

**Published:** 2024-10-02

**Authors:** Adrianna Graja, Mateusz Gumieniak, Maciej Dzimira, Tymon Janisz, Agnieszka Krakos

**Affiliations:** Department of Microsystems, Faculty of Electronics, Photonics and Microsystems, Wroclaw University of Science and Technology, Janiszewskiego 11/17, 50-372 Wroclaw, Poland

**Keywords:** Microfluidics, Lab-on-chip, Passive micromixer, 3D printing, CubeSat, Biological nanosatellite

## Abstract

Nanosatellites of CubeSat type due to, *i.a*., minimized costs of space missions, as well as the potential large application area, have become a significant part of the space economy sector recently. The opportunity to apply miniaturized microsystem (MEMS) tools in satellite space missions further accelerates both the space and the MEMS markets, which in the coming years are considered to become inseparable. As a response to the aforementioned perspectives, this paper presents a microfluidic mixer system for biological research to be conducted onboard CubeSat nanosatellites. As a high complexity of the space systems is not desired due to the need for failure-free and remotely controlled operation, the principal concept of the work was to design an entirely passive micromixer, based on lab-on-chip technologies. For the first time, the microfluidic mixer that uses inertial force generated by rocket engines during launch to the orbit is proposed to provide an appropriate mixing of liquid samples. Such a solution not only saves the space occupied by standard pumping systems, but also reduces the energy requirements, ultimately minimizing the number of battery modules and the whole CubeSat size. The structures of the microfluidic mixers were fabricated entirely out of biocompatible resins using MultiJet 3D printing technology. To verify the functionality of the passive mixing system, optical detection consisting of the array of blue LEDs and phototransistors was applied successfully. The performance of the device was tested utilizing an experimental rocket, as a part of the Spaceport America Cup 2023 competition.

**Graphical abstract:**

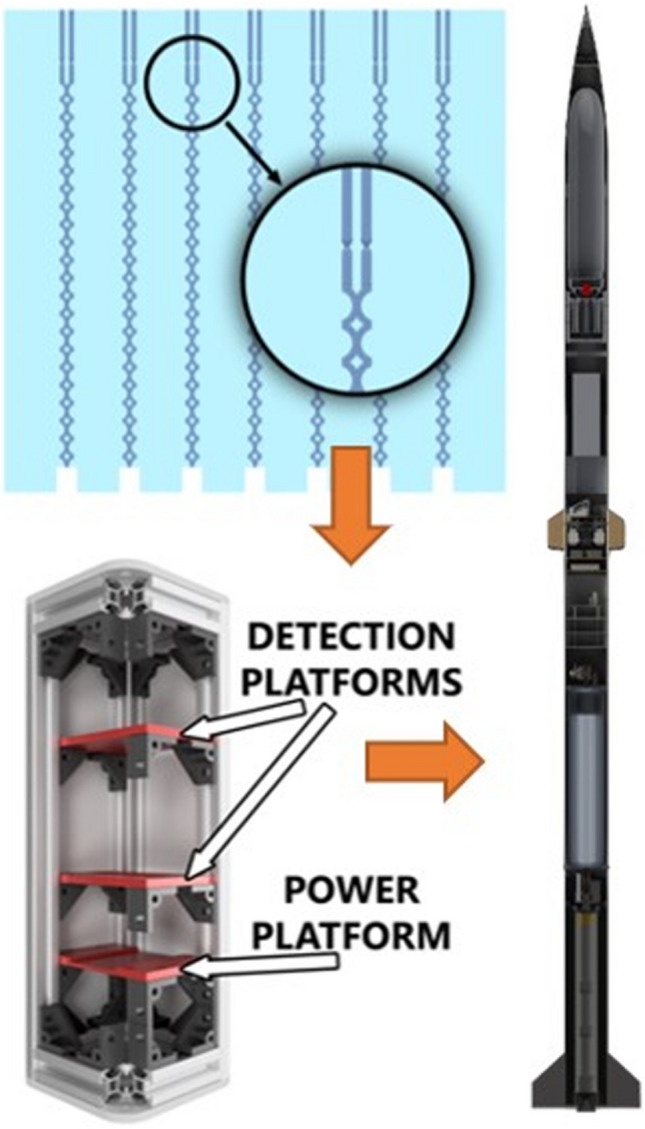

## Introduction

The matter of miniaturized, self-contained, and zero-energy components is increasingly raised by NASA and ESA, as the response to ambitious mission roadmaps, requiring minimized operational systems and costs. Thus, MEMS-based, as well as microfluidic tools, are becoming inseparable elements of the space instruments, mainly nanosatellite payloads, e.g., GeneSat-1, PharmaSat, O/OREOS, SporeSat, EcAMSat, or BioSentinel [1, 2].

For instance, during the first biological CubeSat mission—GeneSat-1 (2006), a 12-well microfluidic card was used to study the culture of *Escherichia coli* and its drug susceptibility. The card was characterized by an optical-quality acrylic face with simultaneous gas permeability, and it was linked to a 15 mL growth medium bag via a solenoid microvalve. Each well was equipped with a 3 W blue LED for fluorescence and a 2.3 mW green LED for optical density measurements [3–5]. Similarly, PharmaSat mission (2009) was focused on on-chip microbial investigation, and a 48-well microfluidic card was provided to assure the culturing of yeasts. Based on the optical absorbance spectra, the colony growth in a form of turbidity measurements was evaluated [6]. In the case of the EcAMSat (2017), it was majorly related to both GeneSat-1 and PharmaSat missions. The payload was adapted from the PharmaSat systems, whereas, biological experimentation was connected to the antibiotic resistance of *E. coli* in microgravity conditions. The payload contained mostly lab-on-chip and MEMS/MOEMS components, i.e., multi-well microfluidic card, micropumps, bubble traps, tubing, as well as LED excitation diodes and optical spectrometer [7, 8].

On the other hand, currently being conducted BioSentinel mission (2022) seems to have the most complex microfluidic system of all mentioned. The payload utilizes 18 polycarbonate fluidic cards, each with 16 wells, for a total number of 288 wells. The cards were designed with multiple layers, incorporating filter membranes to prevent cross-contamination, microchannels for nutrient and waste flow, as well as heating elements to foster yeast growth. Each stack of cards includes optical source and detector boards for monitoring purposes. The fluidic cards are mounted onto manifolds and connected to an array of tubing, reagent bags, pumps, bubble traps, calibration cells, and electronic boards. The system is fully autonomous, enabling controlled rehydration of yeast cells and continuous monitoring of the culturing process [9–12].

Although the laboratory payloads dedicated to CubeSat missions with *life science* focus are more and more diversified, to the best authors’ knowledge, none of the experimental units proposed to date have used entirely passive microfluidics, at which the basic units, e.g., sample injection system, could be limited or even omitted. As the space mission objectives are becoming increasingly innovative and much attention is being paid to assure implementing of multiple experimental tasks simultaneously, a simplified hardware and electronic assistance is necessary [13–15].

As a response to this issue, this paper presents the microfluidic, entirely passive mixer dedicated to biological research performed on CubeSat type nanosatellites. The principle operation of the micromixer is based on the inertial force generated by rocket engines during rocket launch; thus, the sample injection and mixing do not require additional equipment. The structure of the micromixer was fabricated in a form of lab-on-chip instrument, in which the microfluidic valves use capillary forces to hold the liquid in a dedicated reservoir until the rocket takes off. The force of rocket launch causes the liquid to be released from the valves and provided to the mixer microchannels, designed as zig-zag and split-and-recombine (SAR) types. The solution proposed herein is compact and decreases the risk of mission failure, since mixing process does not depend on electronic and/or programmatic actuation. Such an approach can be especially important in the further development of the biomedical space-oriented experiments, where effective mixing of samples and limited electronics assistance is needed.

The passive mixing system proposed in this paper was adapted to a small rocket payload to verify its operation in real flight conditions campaign (Spaceport America Cup 2023). Development of the micromixer, detection system, as well as the whole payload structure are presented in detail in the following parts of the paper.

## Materials and methods

**Fig. 1:**
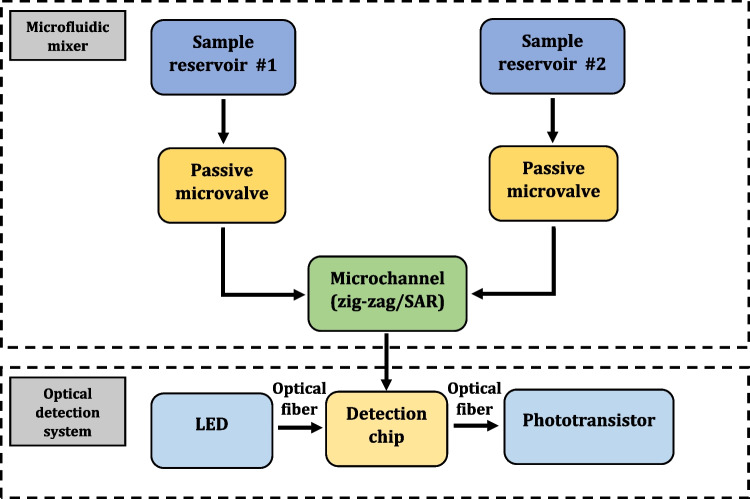
Microfluidic mixer—main components and operational block diagram

The scheme of the microfluidic mixer is shown in Fig. 1. The main components of the mixer are sample reservoirs, microvalves, and microchannels. As mentioned earlier, two types of microchannels were proposed—zig-zag and SAR. These particular shapes were chosen since, based on the recent subject literature [16], they can be considered the most effective when the mixing phenomenon is induced by chaotic advection. Simultaneously, chaotic advection is responsible for sample mixing during turbulent flows described by high Reynold’s number, occurring for instance during the rocket launch.

The assessment of mixing effectiveness is based on the optical detection, which seems the most popular and failure-free approach, utilized typically in the payloads provided by NASA/ESA. Transmitting and receiving multimode poly(methylmethacrylate) (PMMA) fiber optics (core diameter: 0.75 mm) were used to illuminate the sample with the blue LED light (emission wavelength: 470 nm) and obtain the absorbance spectra as phototransistor read-outs (detection range: 400 nm–1100 nm), Fig. 1.

### Microfluidic mixer—design and technology

**Fig. 2:**
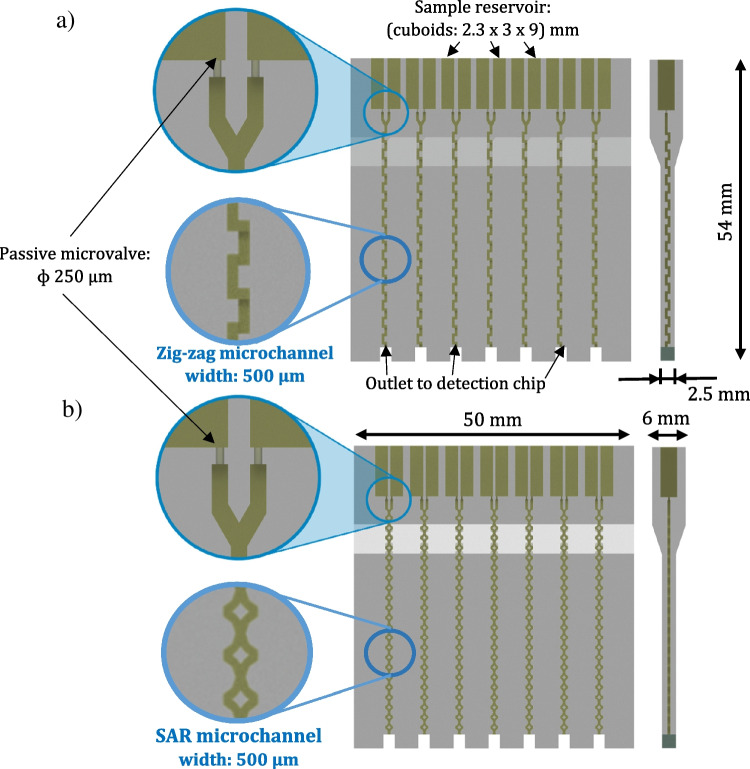
Schemes of the microfluidic mixers with the interesting dimensions: **a** zig-zag, **b** SAR

The schemes of the microfluidic mixers are presented in Fig. 2. As mentioned earlier, active structures that require external energy sources are not the best choice for the space applications; thus, passive micromixers were selected for our research. Passive micromixers base on the microchannels geometries, which special topology is used to provide local turbulence and enhance the mixing process [17, 18]. They are considered much easier to fabricate in comparison to the active micromixers; thus, current development and applicability of these structures are expanding year by year. According to the literature reports [19, 20], the mixing process in passive micromixers is mainly provided by molecular diffusion and chaotic advection. Zig-zag types, next to the meanders, seem the most popular solutions that operation principle relies on laminar flow disturbances due to the constant stream twist. These micromixers work at relatively high Reynolds numbers, which additionally justify their utility in high acceleration environments. On the other hand, SAR micromixers, apart from the chaotic advection, utilize splitting and recombination mechanisms (in other terms, lamination). Geometries of the SAR micromixers split and fold the fluids in a sequential way, improving the molecular diffusion. Effective mixing can be achieved herein by increasing interfacial area exponentially and simultaneously decreasing the diffusion length. SAR structures were confirmed to be applicable over a wide Re range, and a variety of these micromixers are described in the literature [21, 22].

The designs of the micromixers were prepared using Autodesk Inventor, a 3D Computer-Aided Design (CAD) software (Table 1). For both of the micromixers—zig-zag and SAR types, similar dimensions for sample reservoirs and valves were assumed. In the case of the valve fabrication, a special attention was paid to select the most appropriate geometry and diameter of the necking region [5]. Ultimately, an hourglass shape was proposed with the 250-μm diameter of the necking region expanding to the microchannel of 500-μm width. In order to increase the reliability and trustworthiness of the experiments, an array of the micromixers was proposed, and a single lab-on-chip contained seven such structures.

**Table 1:**
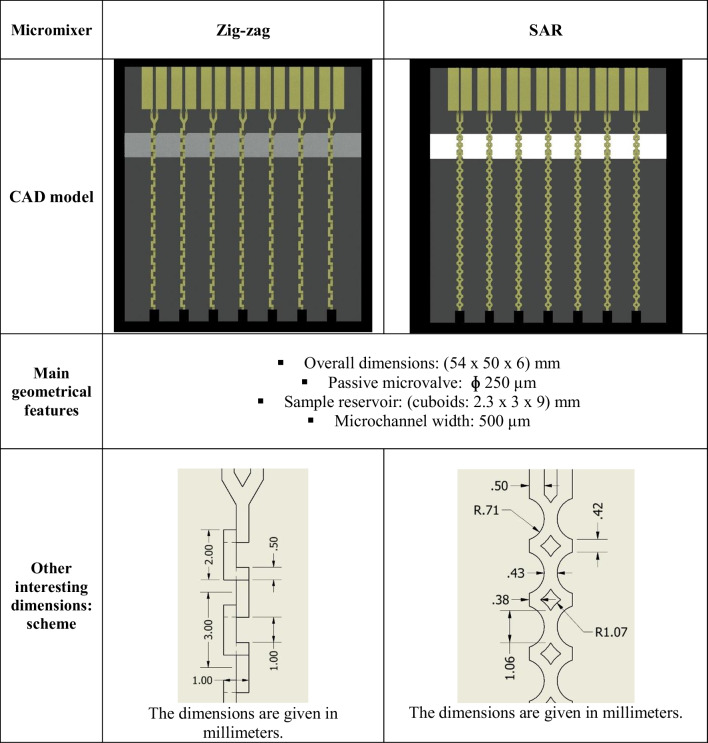
Zig-zag and SAR micromixers—main features and characteristic dimensions

As the idea of rapid prototyping of microfluidic structures is very common nowadays, it was assumed to employ a multi-jet 3D printing technology (printer model: ProJet 3510 SD) with VisiJet Crystal as the build material. The printing process was conducted in XHD (eXtreme High Definition) mode, ensuring a resolution of 750 × 750 × 1600 DPI (xyz), with a single layer thickness of 16 µm. Technically, this method can allow for the reproducible printing of microfluidic channels with widths down to 200 µm. According to the literature reports [23–27], high surface quality of the structures, without any pores or interlayer gaps which could alter flow characteristics, can be achieved herein.

As for the valves utilizing capillary forces, the wettability of the material surface is a crucial parameter; prior to the printing of the micromixers, contact angle measurements of the bare structures fabricated utilizing the aforementioned multi-jet printer were conducted using the sessile drop method. A drop of distilled water was placed on the surface of the printed material, and the angle was determined using dedicated software. Measurements were repeated five times for each sample, each time examining a different spot on a surface. The tests showed that after standard post-processing procedures, surface is hydrophobic, with a mean contact angle of 95.2°. These findings confirmed high utility of demonstrated herein material and technological approach to achieve reliable microfluidic passive stop valves [28].

Next, basic calculations, as well as numerical simulations concerning the performance of the structures, were conducted. At first, the distribution of the forces was defined for both the static laboratory conditions and during the rocket flight. Herein, capillary valve pressure (*P*_*k*_) and opposing inertial forces (*F*) were indicated according to the formulas (1), (2), (3) [28–30]:

1 $${P}_{k}=\frac{2\gamma \times cos\Theta }{r}=\frac{2\times 0.0728\frac{N}{m}\times 0.73}{125\times {10}^{-6}m}=850.3 Pa$$

where:

- γ: surface tension, for water = 0.0728 $\frac{N}{m}$

- $cos\Theta$: cosinus of the contact angle = cos (95°) = 0.73

- $r:$ radius of the necking region = 125 × 10^−6^ m

2 $${F}_{1}=m\times g\;\left(static conditions\right)$$

3 $${F}_{2}=m\times (a+g) \;\left(flight conditions\right)$$

where:

- $m$: mass of the water sample = $V\times \rho$ = $61.91\times {10}^{-6}$ kg

 Since:

$$V=2.3 mm \times 3 mm \times 9 mm=62.1\times {10}^{-9} {m}^{3}$$

$$\rho =997\frac{kg}{{m}^{3}}$$

- a: acceleration of the rocket $=50 \frac{m}{{s}^{2}}$ (based on the available data)

- g: gravity acceleration $$=9.81\frac{m}{{s}^{2}}$$

$${F}_{1}=61.91\times {10}^{-6}\times 9.81=6.07\times {10}^{4}N$$

$${F}_{2}=61.91\times {10}^{-6}\times (50+9.81)=3.70\times {10}^{-3}N$$

Based on the formula (4) and the assumptions that in static conditions the water sample interacts with the cross section of the whole water reservoir, whereas during the rocket flight this is the necking region (leaky area) that plays the pivotal role, the relevant calculations concerning the pressure opposing the capillary forces can be done:

4 $$P=\frac{F}{A}$$

$${P}_{1}=\frac{F1}{A1}=\frac{6.07\times {10}^{-4} }{6.9\times {10}^{-6}}=87.97\;Pa$$

$${P}_{2}=\frac{F2}{A2}=\frac{3.7\times {10}^{-3} }{4.9\times {10}^{-8} }=75 510.2\;Pa$$

where:

- A_1_ $$=a\times b=2.3\times 3 \times {10}^{-6}=6.9\times {10}^{-6}\;{m}^{2}$$  

- A_2_ $$=\Pi {\times r}^{2}=3.14\times {\left(125\times {10}^{-6}\right)}^{2}=4.9\times {10}^{-8}\;{m}^{2}$$  

Thus:

$${P}_{1}\ll {P}_{k}$$

$${P}_{2}\gg {P}_{k}$$

which confirms that the geometry of the micromixers, especially the necking region, is appropriately designed and the structures should not ensure the water sample to leave the reservoir before the rocket launches.

**Fig. 3:**
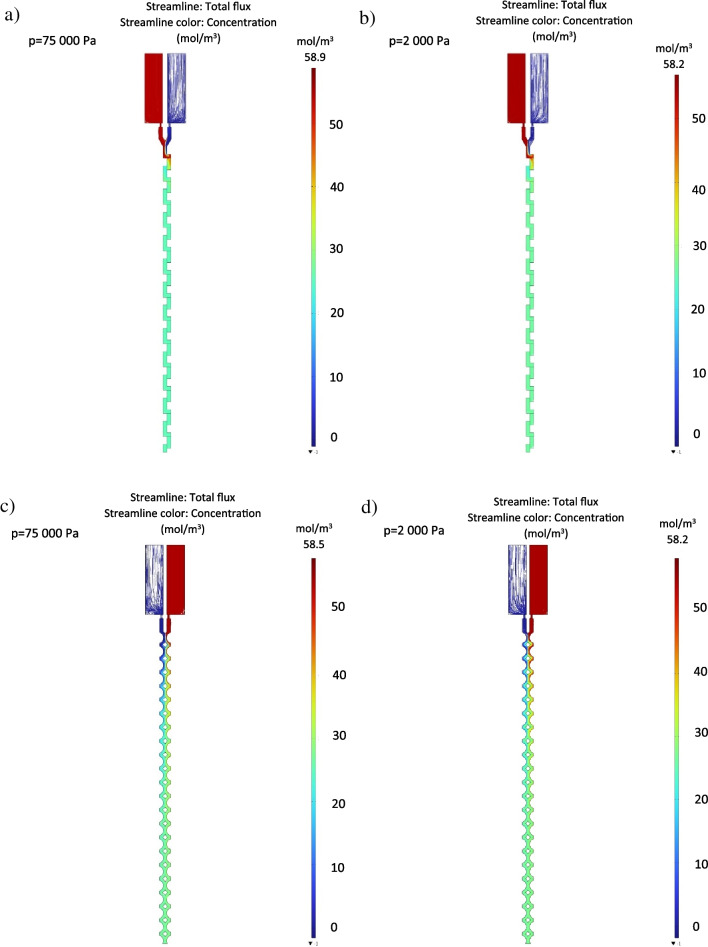
Simulations of the mixing in the designed structures: **a** zig-zag (*P* = 75,000 Pa), **b** zig-zag (*P* = 2000 Pa), **c** SAR (*P* = 75,000 Pa), **d** SAR (*P* = 2000 Pa). As shown, mixing of the samples occurs fast, after circa one (zig-zag) and six (SAR) mixing units [20]

Numerical simulations concerning the samples mixing in the designed structures were conducted in COMSOL Multiphysics 4.3 software, utilizing the physical interfaces “Laminar Flow” and “Transport of Diluted Species” [31, 32]. Mesh quality was set to “Normal”. The boundary conditions regarding the microflow were assumed according to the provided calculations. Thus, the pressures exceeding the capillary forces (2000 Pa) and simultaneously imitating rocket launch (75,000 Pa) were defined. Fluid flow through the microfluidic channel outlets was read back from simulation output for each given pressure. The examples of the micromixers performance are shown in Fig. 3.

Next, Reynolds number (*Re*) was calculated according to the formula (5), where the high value corresponds to the micromixer application range:

5 $$Re= \frac{u\times l}{v}$$

where:

- *u*: flow velocity—1.7 m/s (defined based on the velocity graph from Comsol for the maximum pressure of 75 kPa).

- *l*: characteristic length—500 µm (microchannel width).

- *ν*: kinematic viscosity of the fluid (water)—1.0034 mm^2^/s

$$Re= \frac{u\times l}{v}=\frac{1.7\times 500\times {10}^{-6}}{1.0034\times {10}^{-6}}=\sim 850$$

**Fig. 4:**
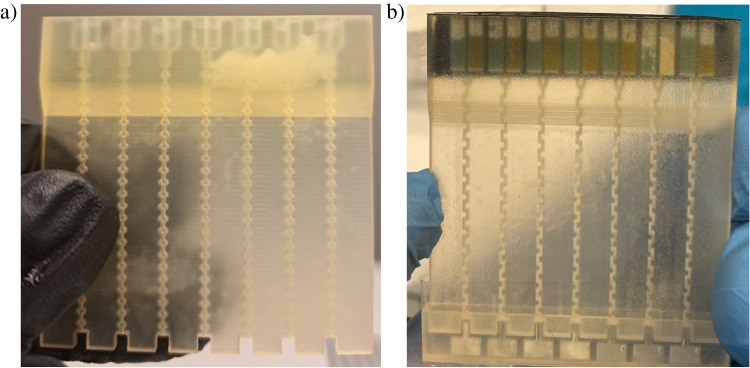
Microfluidic mixers at a glance: **a** SAR, **b** zig-zag with sample reservoirs filled with blue and orange dyes to confirm valves tightness in the static laboratory conditions

As mentioned, the microfluidic mixers were fabricated utilizing multi-jet 3D printing technology (printer model: ProJet 3510 SD) and biocompatible materials (ViSiJet S300 and ViSiJet M3 Crystal), Fig. 4. After the printing process, the structures were cleaned according to the procedure described elsewhere [25] and tested.

**Fig. 5:**
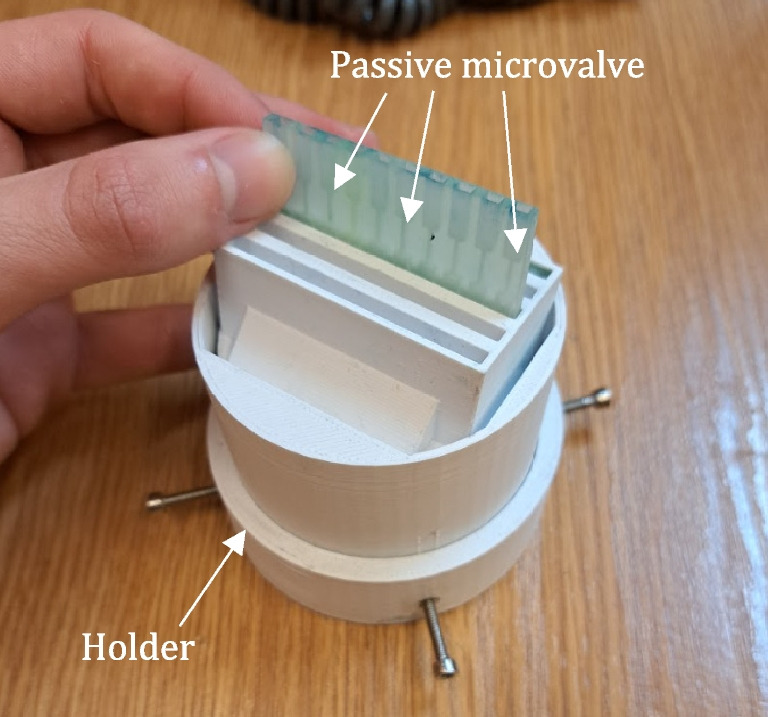
Leakage tests of the micromixer capillary valves: the lab-on-chip with a water-dye sample (solution of methylene blue) injected into the reservoirs was inserted into a holder. In the case of the leaky valves, the sample would fall directly onto the cotton swab placed in the holder, leaving the blue stain

The tests of the micromixers were conducted in ambient temperature, as well as in 4 °C to verify their operation in higher humidity. As a result, it was observed that the microvalves do not release the sample, and capillary forces are strong enough to keep the water-dye solution in the reservoirs in the static conditions (Fig. 5). For the appropriate protection of the samples, the upper parts of the reservoirs were covered with a specialized foil, in which small air holes were made using a needle.

Next, the ground tests showing that the microfluidic mixers can allow for the appropriate mixing of the samples were conducted. To confirm that, we have used the dyes which are well known for the diverse biological studies—methylene blue (Merck KGaA, Darmstadt, Germany) and methyl orange (Merck KGaA, Darmstadt, Germany), as well as which can provide an unambiguous information about the final mixing quality (green hue as a mixing product).

According to the literature reports, methylene blue can determine the viability of cells (e.g., yeasts) by staining the dead cells which then appear as dark blue cells [33, 34]. On that basis, our microfluidic mixer could be used for the future space mission which aims at the evaluation of the development of cell cultures in microgravity conditions. For instance, cells immersed in the culturing buffer could be mixed with the blue dye during the rocket launch and, next, introduced into the culturing chamber for the long-term space exposition.

On the other hand, methyl orange is a dye which is used typically as a pH indicator [35, 36]. Similarly as in the case of methylene blue, it could be applied for the evaluation of the cell culturing experiment during the CubeSat mission, namely to assess the potential degradation of cell culturing medium in time and, thus, condition of cells in microgravity.

**Fig. 6:**
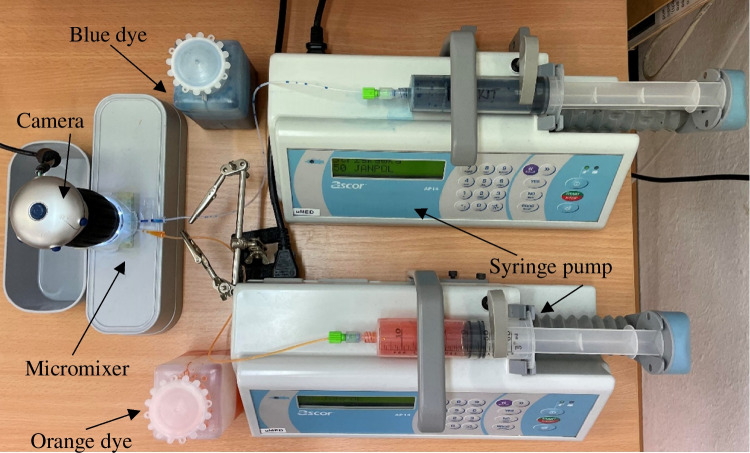
Measurement setup for the verification of micromixers mixing capability at a glance

In the tests showing the mixing capability, a simple measurement setup ensuring the injection of the dyes into the micromixers sample reservoirs was used (Fig. 6). Laboratory syringe pumps (AP14, Ascor, Poland) were employed to breach the capillary pressure barrier and provide the overflown of the stop valves.

**Fig. 7:**
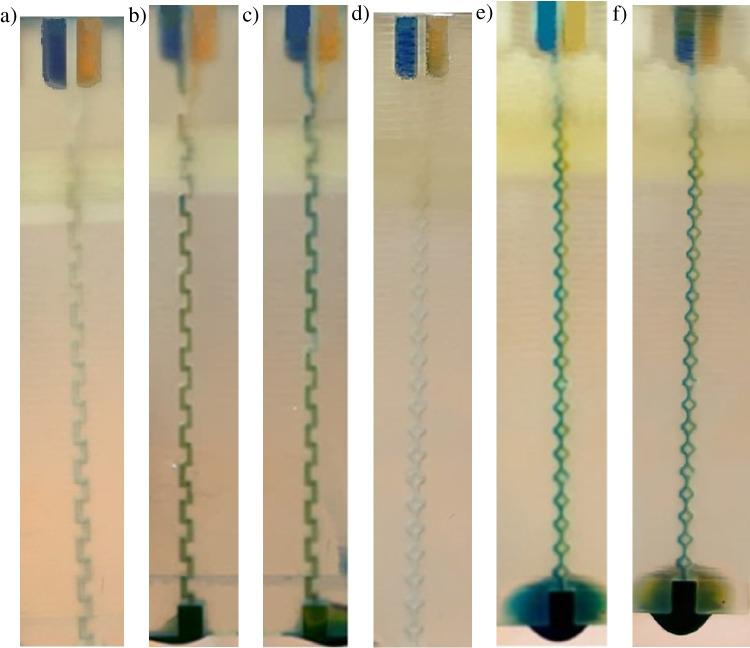
Mixing of the dyes in the microfluidic mixers: **a** zig-zag microchannel 1 (850 Pa, 64.5 ml/h), **b** zig-zag microchannel 2 (2000 Pa, 112 ml/h), **c** zig-zag microchannel 3 (75,000 Pa, 846 ml/h), **d** SAR microchannel 1 (850 Pa, 84.8 ml/h), **e**) SAR microchannel 2 (2000 Pa, 154 ml/h), **f** SAR microchannel 3 (75,000 Pa, 1304 ml/h)

Based on the calculated data, as well as Comsol numerical simulations provided in the manuscript for the selected pressure values (2000 Pa, 75,000 Pa), it was possible to read out the interesting volume flow rates (Table 2). Next, these flow values were set on the syringe pumps to provoke the micromixing process. The confirmation of appropriate performance of the stop valve for the capillary pressure equaling to 850 Pa that keeps the sample within the reservoirs was also made. The results of these experiments are shown in Fig. 7.

**Table 2:** Flow rates corresponding to the selected pressure values—collected based on the Comsol data

Pressure [Pa]	Flow [ml/h]	
	Zig-zag	SAR
**75,000**	**846**	**1304**
50,000	681	1052
20,000	415	631
10,000	284	422
5000	192	276
**2000**	**112**	**154**
1000	71.9	95.5
**850**	**64.5**	**84.8**
500	44.6	56.4
200	21.8	25.6
100	11.8	13.2
50	6.06	6.71
20	2.45	2.69
10	1.23	1.35

As shown in Fig. 7, both of the micromixers can mix blue and yellow dyes to obtain a green dye as a final product. Nevertheless, it seems that zig-zag provides the green mixture faster, circa after four to five mixing units, while SAR after 10–12 segments. These observations are consistent with the numerical simulations presented in the manuscript, at which SAR mixing capability was also less effective. Probably, geometry of the SAR micromixer is not fully optimized, and “split” and “recombine” elements should be designed more carefully [20]. In this way, the length of the microchannels could be also reduced for the future tasks which would favor the matter of structures cleaning procedures. Similarly as shown in the simulations, the value of the pressure breaching the capillary pressure (2000 Pa or 75,000 Pa) does not have any significant meaning in the context of mixing time. Although the optical clarity of the material used for the micromixer fabrication is not excellent, it can be easily evaluated (even with the naked eye) that the green mixture can be obtained.

### Optical detection system—design and operation

**Fig. 8:**
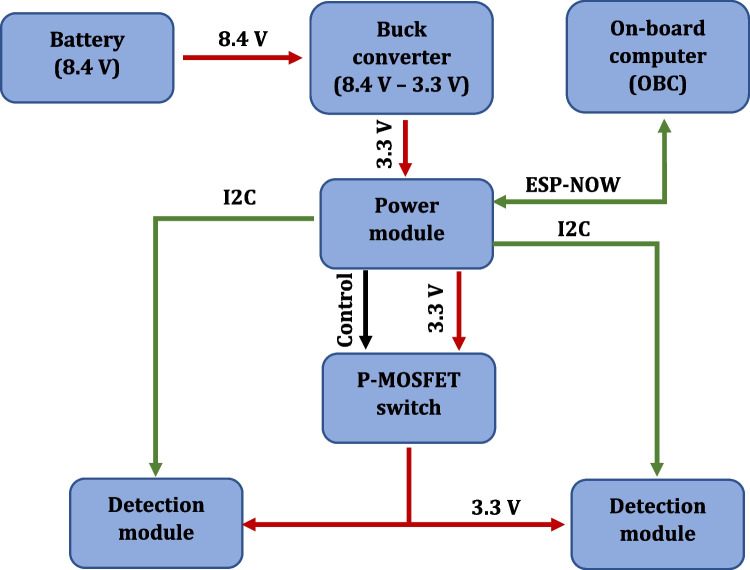
Optical detection system—main components and operational block diagram

To confirm the quality of the mixing, the optical detection system was developed to measure the absorbance of the sample over time. To simplify the connection of the system to the On- Board Computer (OBC) of the rocket, ESP32 was used as the microcontroller unit (MCU) (OBC also uses ESP32). To enhance the system reliability, a power (communication) module and a detection module were separated in the design (Fig. 8).

**Fig. 9:**
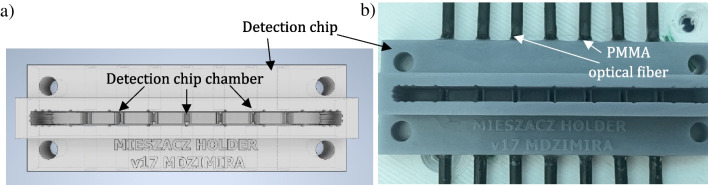
Detection chip: **a** model (designed in the Autodesk Inventor software), **b** 3D printed structure (printed utilizing self-developed FDM 3D printer) with optical fibers assembled

The power module communicates with the OBC and, depending on the current state of the rocket, turns on power to the detection modules and switches on data recording. The detection module controls LEDs to which the PMMA optical fibers are attached. Light is transmitted to the detection chambers filled with mixed sample in the detection chip (Fig. 9) and, next, reaches the phototransistors. The voltage value on the phototransistors in the voltage divider circuit is written on the SD card and into the MCU’s flash memory.

**Fig. 10:**
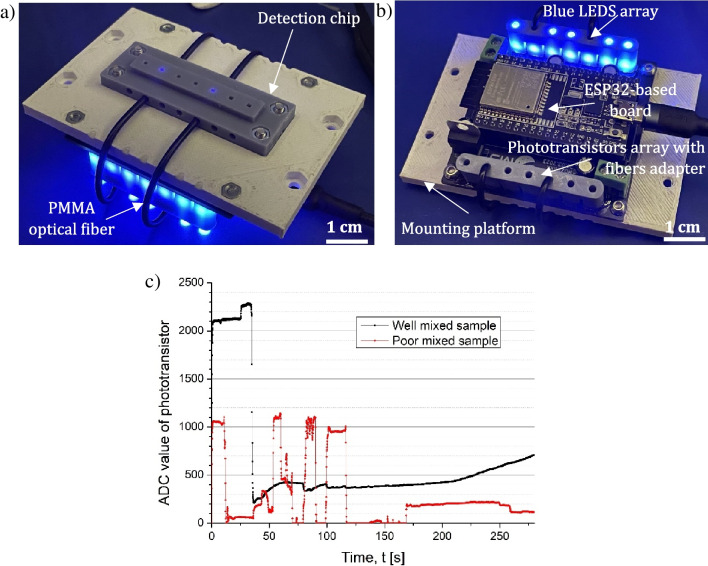
The tests of the detection chip assembled with the optical detection board: **a** bottom view, **b** top view, **c** measurement data

The detection chip assembled with the detection board is shown in Fig. 10. In order to verify the operation of the optical detection system, the samples (water-flour solutions) were prepared (mixed by hand) to achieve appropriately and poorly mixed solutions. Next, the samples were injected into the detection chip to confirm the changes in detection read-outs. Based on the measurement data (Fig. 10), significant differences in absorbance charts could be observed, demonstrating suitable performance of the optical detection system.

### Microfluidic mixer—assembly in the payload structure

**Fig. 11:**
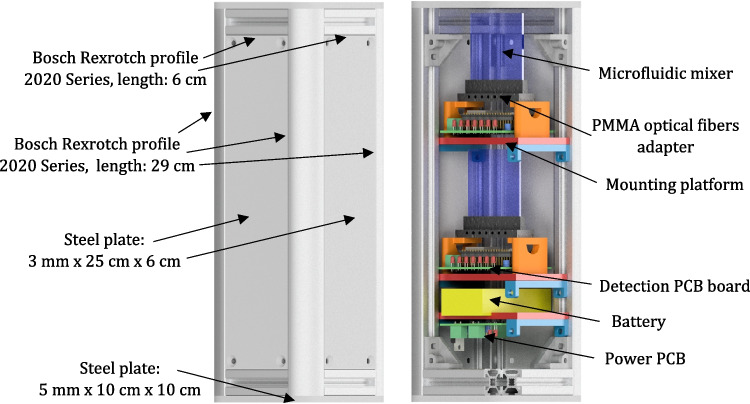
Microfluidic mixer payload—CubeSat model (on the left exterior, on the right interior)

For the integration of the micromixer system in the rocket, a dedicated frame provided in the form of a 3U CubeSat (30 cm × 10 cm × 10 cm) was designed (Fig. 11). The structure was aimed to fit two mixing systems (one for the zig-zag, the second for SAR) and ensure experiment multiplication during a single mission concept.

**Fig. 12:**
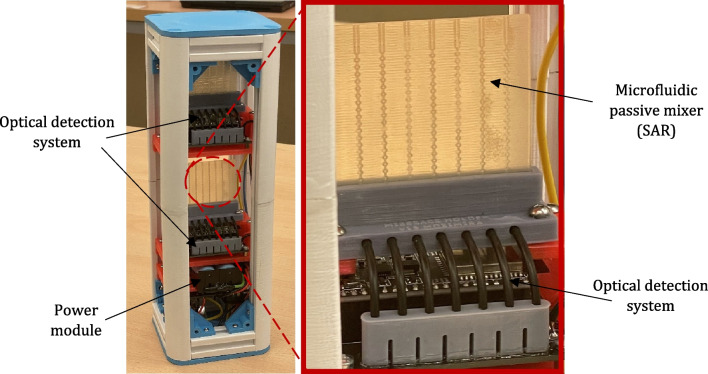
3D printed prototype of the microfluidic mixer assembled into the CubeSat structure

Next, the prototype of the payload was 3D printed utilizing FDM printer (similarly as for the detection chip), entirely from PLA material. Power and detection platforms were inserted into the payload v-slots to check the structure functionality prior to the mission (Fig. 12).

The integration tests of the structure were provided to check if all the payload modules communicate with the rocket correctly. For instance, communication with the rocket OBC (On-Board Computer), PWM control, and recording the voltage signal on the phototransistor to the SD card and FLASH memory were tested. Ultimately, all the communication and detection tasks were provided, showing excellent performance of the micromixer system.

**Fig. 13:**
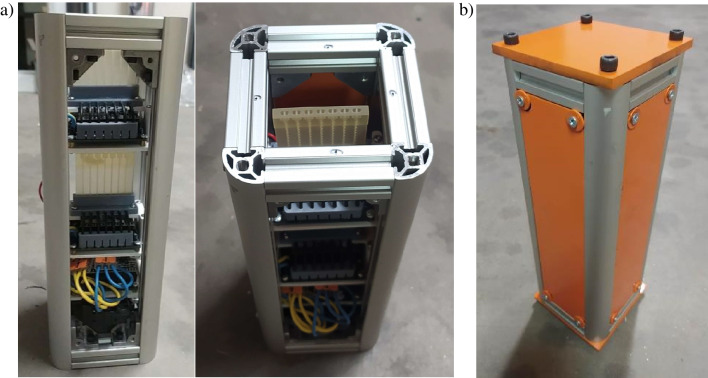
CubeSat at a glance: **a** front and top views, **b** final closed structure

As the 3D printed CubeSat prototype showed that the structure works appropriately, the final assembly could be done, based on the commercially available aluminum BOSCH profiles. To fit the payload inside Student Researched and Developed (SRAD) rocket, a round plate of DIBOND composite with diameter of 15 cm was used. The final structure of the payload is shown in Fig. 13.

## Results and discussion

**Fig. 14:**
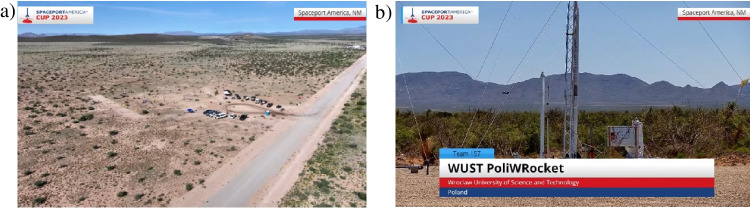
Spaceport America Cup 2023: **a** competition site, **b** rocket with the payload on-board prior to the launch

The tests of the microfluidic mixer payload were conducted during the Spaceport America Cup, the rocketry competition held on the 19.06–24.06 in Jornada del Muerto desert in the state of NM, USA, 2023. The payload was integrated with the experimental rocket—Aurora, constructed by PWr In Space scientific community from Wroclaw University of Science and Technology (WUST). The rocket was categorized for the flight at an altitude of 10.000 feet, Fig. 14.

**Fig. 15:**
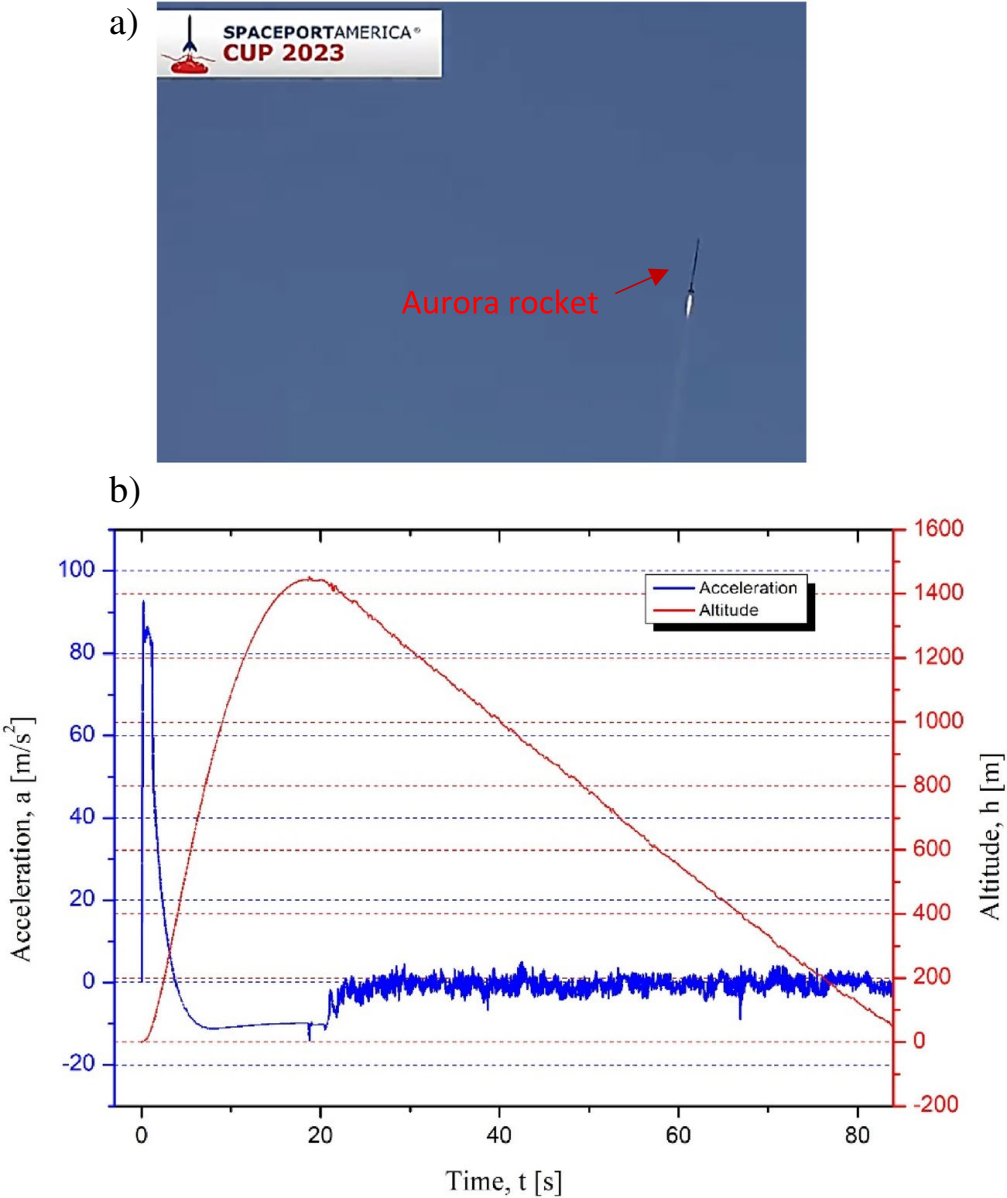
Rocket launch: **a** Aurora rocket in the air, **b** data acquired from the rocket OBC regarding altitude and Z axis vertical acceleration

Unfortunately during the competition, due to the external factors beyond our control (wind, organizational problems), there was the substantial delay in launch of the rocket. For this reason, the time between the sample injection to the microfluidic mixer system and rocket launch (Fig. 15) was about 72 h.

**Fig. 16:**
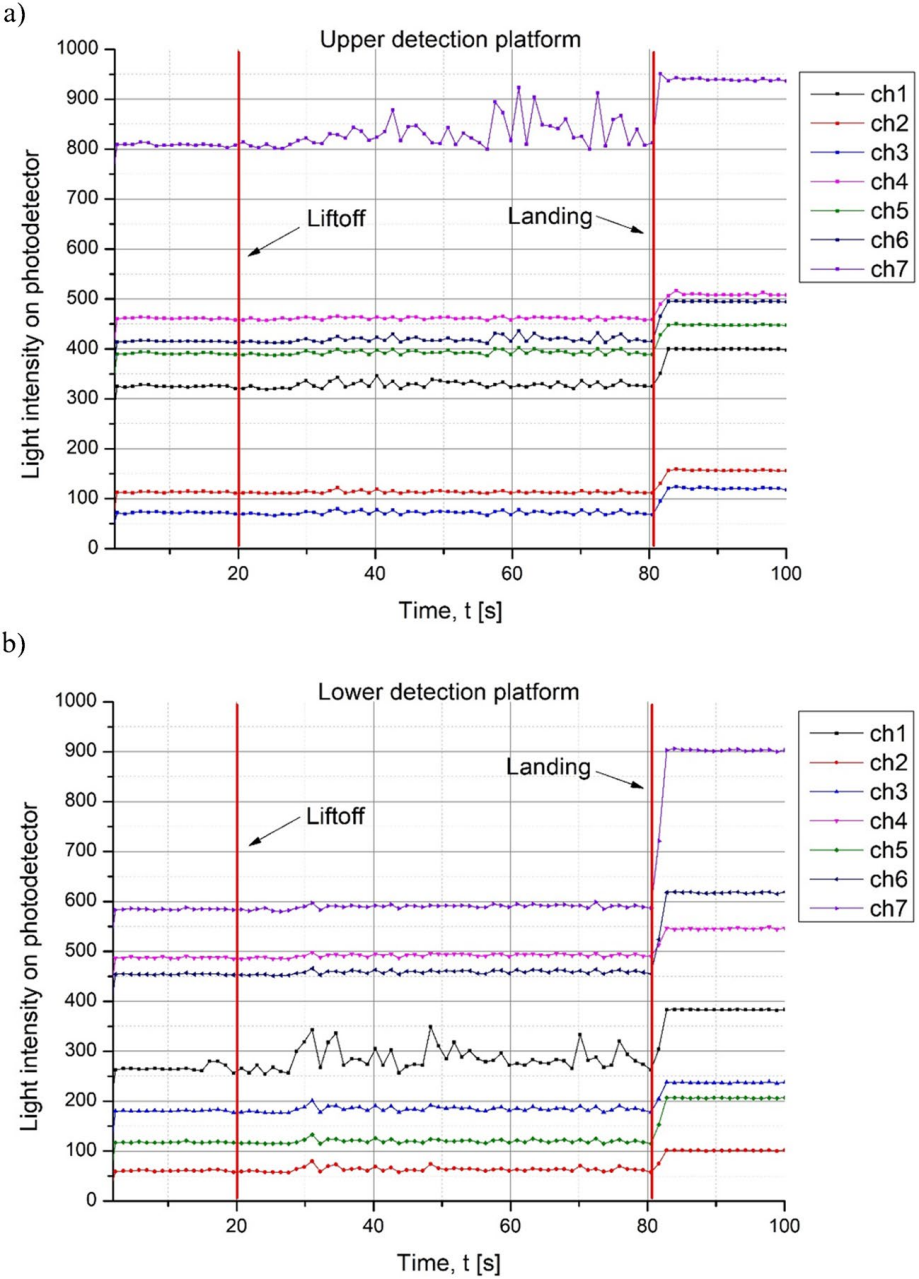
Optical detection system response: **a** upper detection platform data, **b** lower detection platform data

Although the primary goal of the tests was to verify if all the rocket-payload subsystems work and communicate appropriately in real-flight conditions, the experiments related directly to passive microfluidic mixing implemented in the payload could not be conducted properly. Due to the aforementioned, unforeseen circumstances, most likely while waiting for the launch in high temperatures (over + 40 °C), the content of the sample reservoirs could have evaporated and penetrated the microfluidic valves unexpectedly, before the main competition started. Therefore, the detection system registered solely a change in light transmission through the detection chamber when the rocket hit the ground (landing phase). This can be the result of potential displacement of the platform including the optical fibers. Probably, due to the hit, optical fibers shifted slightly, changing the indications visible on the graph. In addition, the optical fibers were glued with the adapter which may provide some flexibility and result in change of the read-outs when the system faces such an impact. On the other hand, small changes of the light intensity observed during the flight phase (20–80 s), especially for microchannel 7 (platform 1) and microchannel 1 (platform 2), are probably connected to the background noise. These observations are similar for both detection platforms (Fig. 16).

As the laboratory tests and observations provided on the ground have confirmed that the microfluidic mixers exhibited appropriate performance, the issues connected directly to the mechanical and thermal protection should be improved majorly. The application of a temperature control system within the payload, along with fully hermetic sealing, is essential for protecting samples from temperature fluctuations and should be implemented in future target missions. While in this specific experimental scenario, vibrations facilitated the desired movement of liquid through the microvalve into the micromixer microchannel, it remains crucial to ensure proper connections and assembly of the payload structure. The unique nature of biochemical space experiments necessitates specialized materials and technological approaches to prevent sample evaporation. Additionally, careful design of components and their precise alignment is imperative. Similarly, dedicated yet straightforward electronic control units are essential for ensuring reliable, fault-free operation.

## Conclusion

To sum up, the solutions of multi-jet, 3D printed passive micromixers to be used as a laboratory payload in space missions were shown. The microfluidic structures were fabricated out of biocompatible polymers to enhance their application in biochemical experimentation. As the need for minimized weight and size of the space instrumentation is notably visible these days, we proposed a simplified design that can reduce complexity of microfluidic systems in biological payloads in future mission concepts. Inertial force generated during the rocket launch was used to initialize the mixing process of two samples in zig-zag and SAR type microchannels. Microfluidic valves utilizing capillary forces maintained the analytes in mixer reservoirs until the overload occurred, which was confirmed in the ground-based tests. Thanks to the optical detection system that bases on absorbance measurement in time, the quality of the mixing process could be indicated. To the best authors’ knowledge, this is the first such a solution, and the concept of the passive, inertial-based microfluidic mixer for space applications has not been presented in the literature to date. Unfortunately, verification tests performed during the Spaceport America Cup 2023 were not successful, and ultimately, the mixing process could not be registered. Nevertheless, working of the payload with the mixer onboard was checked and showed perfect performance, indicating appropriate assembly of the whole electronic and mechanical components within. Probably, better thermal and mechanical insulation could protect the samples from evaporation in harsh, high temperature conditions and provide positive experimental results. These and other modifications of the mixing system will be the subject of the further works.

## Data Availability

No datasets were generated or analysed during the current study.
